# Disseminated Intravascular Coagulation in Sepsis and Associated Factors

**DOI:** 10.3390/jcm11216480

**Published:** 2022-10-31

**Authors:** Ikhwan Rinaldi, Mondastri Korib Sudaryo, Nurhayati Adnan Prihartono

**Affiliations:** 1 Department of Epidemiology, Faculty of Public Health, Universitas Indonesia, Depok 16424, Indonesia; 2 Division of Hematology and Medical Oncology, Department of Internal Medicine, Cipto Mangunkusumo National General Hospital, Faculty of Medicine, Universitas Indonesia, Jakarta 10430, Indonesia

**Keywords:** sepsis, DIC, coagulation, infection

## Abstract

Background: sepsis is a life-threatening organ dysfunction caused by an excessive host immunological response to infection. The incidence of sepsis is increasing every year, and sepsis is the primary cause of mortality in intensive care units (ICUs). DIC is a coagulopathy syndrome that causes microvascular and macrovascular thrombosis and increases the risk of bleeding due to consumptive coagulopathy. The pathophysiology of DIC in sepsis is complex, and further research is required to investigate the involved mechanisms and risk factors. Method: this study is a prognostic analysis of a retrospective cohort. Samples were patients diagnosed with sepsis and admitted to Cipto Mangunkusumo National General Hospital from January 2016 to October 2022. Research subjects were followed until occurrence of DIC during sepsis or recovery from sepsis. The research subjects were selected from medical records using a consecutive total sampling approach. The inclusion criteria were patients aged ≥18 years old and diagnosed with sepsis according to qSOFA criteria with a score of 2. The exclusion criterion was an incomplete medical record. Bivariate and multivariate logistic regression analyses were performed to determine which independent variables contributed to the incidence of DIC and obtain the odds ratios (ORs). *p* < 0.05 was considered to indicate a statistically significant difference. Results: a total of 248 patients were included after considering the inclusion and exclusion criteria. Of these, 50 (20.2%) septic patients developed DIC. In the multivariate analysis, albumin ≤2.5 g/dL (OR: 2.363; 95% CI: 1.201–4.649), respiratory infection (OR: 2.414; 95% CI: 1.046–5.571), and antibiotic treatment ≥1 h (OR: 2.181; 95% CI: 1.014–4.689) were associated with DIC development. On the basis of the ROC curve, the area under the curve (AUC) was determined to be 0.705 with 95% CI = (0.631–0.778). Conclusion: in our study, the prevalence of DIC in septic patients was 20.2%. Low albumin, respiratory infection, and antibiotic treatment ≥1 h were found to be risk factors for development of DIC in septic patients.

## 1. Introduction

Sepsis is a life-threatening organ dysfunction caused by an excessive host immunological response to infection [1,2]. The incidence of sepsis is increasing every year, and sepsis is the primary cause of mortality in intensive care units (ICUs) [3,4,5]. According to Dugar et al., sepsis is increasing due to expansion of the elderly population and the escalating incidence of chronic diseases [6].

DIC is a coagulopathy syndrome that causes microvascular and macrovascular thrombosis and increases the risk of bleeding due to consumptive coagulopathy [7]. In DIC, blockages caused by thrombi in microvascular and macrovascular tissues result in tissue ischemia, which can exacerbate organ dysfunction [7]. As a consequence of systemic consumptive coagulopathy, there is a decrease in the level of coagulation factors in body, which results in spontaneous bleeding and simultaneous thrombus formation. Septic patients that develop DIC have a poor prognosis [8,9]. The pathophysiology of DIC in sepsis is complex, and further research is required to investigate the involved mechanisms and risk factors. In addition, septic patients with DIC have a worse clinical condition and are at increased risk of mortality and systemic organ dysfunction. There is currently no history, physical examination, or laboratory component that can accurately diagnose or rule out DIC in a patient. Both gram-negative and gram-positive bacteria may cause DIC, although it is more common in gram-negative bacteria [10,11].

Several criteria for the diagnosis of DIC have been established by the International Society on Thrombosis and Haemostasis (ISTH), the Japanese Ministry of Health Labor and Welfare (JMHLW), and the Japanese Association of Acute Medicine (JAAM). These criteria incorporate laboratory tests for several components such as fibrinogen, prothrombin time, and D-dimer levels. It should be noted that laboratory examination of these components is not routinely carried out in many developing countries, except in tertiary hospitals. In addition, these inspections require are costly and time-consuming, resulting in potential delays in diagnosis or treatment. These criteria also do not include conditions, such as heart failure and liver disease, that are indicated to cause DIC [12,13,14,15,16]. As a result, their use in clinical situations in Indonesia is impractical. In Indonesia, the incidence of sepsis and its associated mortality are quite high [17].

Mortality in sepsis increases if complications arise, such as disseminated intravascular coagulation (DIC) [7,9]. According to a study by Dhainau et al., DIC is a strong predictor of mortality in septic patients independent of the severity of sepsis [9]. There have been various therapeutic developments in sepsis, but very few studies have been conducted on DIC in sepsis [17]. Thus, factors associated with the incidence of DIC in septic patients should be statistically analyzed.

## 2. Method

This study is a prognostic analysis with a retrospective cohort design. Samples were patients diagnosed with sepsis and admitted to Cipto Mangunkusumo National General Hospital, which is a national referral tertiary hospital, from January 2016 to October 2022. Research subjects were followed until DIC occurred during sepsis or recovery from sepsis. The research subjects were selected from medical records using a consecutive total sampling approach.

## 3. Inclusion and Exclusion Criteria

The inclusion criteria were patients aged ≥18 years old diagnosed with sepsis according to qSOFA criteria with a score of 2. qSOFA is a score developed through simplification of SOFA for more rapid diagnosis in a non-ICU setting. A study by Koch et al. showed that the qSOFA score performs sufficiently well compared to more complex models such as SOFA or LODS in a non-ICU setting [18]. The exclusion criterion was an incomplete medical record.

## 4. Variables

The operational definition of DIC refers to the ISTH criteria consisting of decreased platelet levels, elevation of D-dimer or other fibrin degradation products, prolongation of prothrombin time, and abnormal fibrinogen levels.

The independent variables studied were age, sex, hemoglobin level, leukocyte count, neutrophil/lymphocyte count ratio, type of infected organ, solid cancer, hematological cancer, metabolic disease, autoimmune disease, chronic kidney disease, autoimmune therapy, impaired liver function, anti-aggregation therapy, blood culture, time to start antibiotics, renal failure, and heart failure.

Sex of the patients was based on the data registered in the medical record. Age of the patient was the age when the patients were diagnosed with sepsis, categorized as either less than 60 years old or 60 years old and above. Infected organs in this study were categorized into respiratory or non-respiratory organs. In this study, solid cancers referred to are all cancers excluding neoplasms, bone marrow, and lymph nodes. Hematologic cancers included leukemia, multiple myeloma, and lymphoma.

Recorded metabolic diseases were diabetes mellitus (type I or II), dyslipidemia, and hypertension. In this study, liver dysfunction was defined as an increase in AST or ALT more than three times the normal levels or a known history of chronic liver comorbidities such as liver cirrhosis, chronic hepatitis, and fatty liver. Autoimmune diseases included in the study are systemic lupus erythematosus, rheumatoid arthritis, multiple sclerosis, asthma, inflammatory bowel disease, and vasculitis. The definition of heart failure in the study referred to the Framingham criteria, any history of renal failure undergoing hemodialysis acquired from the medical records, or an eGFR under 30 mL/min/m^3^. eGFR was calculated using MDRD equation.

The definitions of hematologic parameters are as follows: anemia was defined as a hemoglobin level under 13 g/dL for males and under 12 g/dL for females, leukocytosis was defined as leukocyte levels above 10,000/mm^3^, and a neutrophil/leukocyte ratio above 9.0 was considered as high.

## 5. Statistical Analysis

Medical record data from 2016 to 2022 were descriptively processed to determine the incidence of DIC in septic patients. Numerical data with a normal distribution are presented as mean and standard deviation. Numerical data with a non-normal distribution are presented as median and minimum–maximum values. Categorical data are presented in the form of percentages.

Bivariate and multivariate logistic regression analyses were performed to determine which independent variables contributed to the incidence of DIC and obtain odds ratios (ORs). *p* < 0.05 was considered to indicate a significant difference. Multivariate analysis was conducted using the backward Wald method. All statistical analysis was conducted using SPSS software version 21 (SPSS Inc., Armonk, NY, USA).

## 6. Result

A total of 248 patients were included after considering the inclusion and exclusion criteria. This included 50 (20.2%) septic patients that developed DIC and 198 (79.8%) septic patients that did not (Table 1). Compared with those that did not develop DIC, patients that developed DIC were younger with a mean age of 49.76 ± 13.97 years old. There was a total of 127 males (51.2%) in the study population. The mean hemoglobin in the group that developed DIC was 8.99 ± 2.68, while the mean hemoglobin in the group that did not develop DIC was 9.83 ± 2.57.

Chronic kidney disease was the most common comorbidity in the study population with a total of 139 (56%) patients. On the other hand, liver disease was observed in 48 (19.4%) patients. The most common site of infection was the pulmonary system in the case of 183 (73.8%) patients (Table 2). For solid and hematological cancers, there were 84 (33.9%) patients and 24 (9.7%) patients, respectively. The most common type of hematological cancers was non-Hodgkin lymphoma (Table 3). The characteristics of patients with sepsis, DIC, and liver disease can be seen on Supplementary Table S1. Meanwhile, characteristics of patients with sepsis, DIC, and hematological cancer can be seen on Supplementary Table S2.

In the bivariate analysis, several variables were found to be statistically significantly associated with DIC development, such as age ≥60 years, hematological cancer, metabolic disease, low albumin, and antibiotic treatment ≥1 h (Table 4). The variable with the highest odds ratio was hematological cancer (OR: 3.286; 95% CI 1.362–7.926). However, after conducting multivariate analysis of all the variables in this study, only three factors were found to be significant (Table 5), namely albumin ≤2.5 (OR: 2.363; 95% CI: 1.201–4.649), respiratory infection (OR: 2.414; 95% CI: 1.046–5.571), and antibiotic treatment ≥1 h (OR: 2.181; 95% CI: 1.014–4.689). The Hosmer and Lemeshow test for multivariate analysis yielded a *p*-value of 0.130, which indicates the model is well-calibrated.

**Figure 1:**
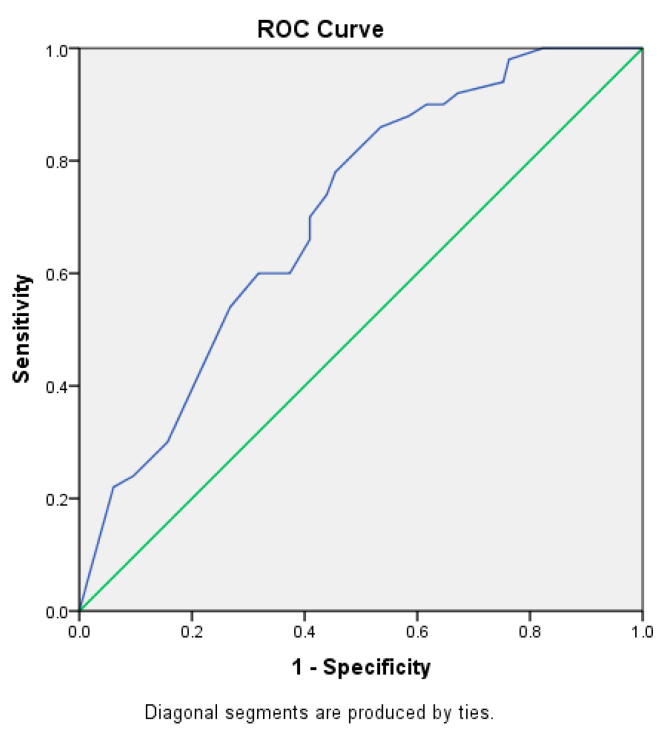
ROC curve. Green line indicates random classifier. Blue line indicates the multivariate model obtained from Table 5.

Using the multivariate model, the ROC curve (Figure 1) was obtained, and the area under the curve (AUC) was determined to be 0.705 with 95% CI = (0.631–0.778).

## 7. Discussion

This study is the first to analyze factors associated with the development of DIC in the context of sepsis. From the analysis of the research results, it was found that low albumin ≤2.5 g/dL, respiratory infection, and antibiotic treatment ≥1 h are significantly associated with the incidence of DIC in septic patients. In this analysis, the three significant variables are risk factors for DIC in septic patients, with an odds ratio of 2.363 for low albumin representing liver dysfunction, 2.414 for respiratory infection, and 2.181 for receiving antibiotic treatment after 1 h or longer.

In our study, we found low albumin level is associated with DIC development in septic patients, although we did not find a significant association between history of liver disease and DIC incidence. However, as albumin is produced by the liver, the level of albumin is a marker of liver function, and low albumin could represent the state of liver dysfunction. Pathophysiologically, liver disease is a risk factor for DIC in septic patients, because the liver produces factors that play a role in coagulation [19,20]. In liver disease, there is a decrease in the levels of both procoagulant factors and coagulation inhibitors, abnormal fibrinogen and prothrombin production, as well as abnormalities in both the quantity and quality of platelets [19,20]. These changes lead to an imbalance between coagulation and fibrinolysis processes, representing potential risk factors for developing DIC. Low albumin may also be caused by CKD.

In a previous case–control study on the incidence of liver disease on DIC in septic patients conducted by Anderko et al. (2022), it was found that 6% (82 of a total sample of 1341) of patients with septic shock had hepatobiliary dysfunction and DIC [21]. It was also shown that the condition was an independent risk factor for patient mortality at 90 days (OR 3.1, 95% CI: 1.4–7.5, *p:* 0.008) [21]. However, there are currently no prospective studies assessing the association between liver function in septic patients and risk of DIC.

A challenge in associating liver disease with DIC in septic patient lies in differentiating coagulative abnormalities post liver diseases as DIC from other hemostatic diseases [22]. In the clinical setting, septic patients without a history of liver disease may also develop liver dysfunction as a consequence of sepsis-associated organ dysfunction. In our study, we addressed these challenges by the use of overt DIC criteria (prothrombin time, D-dimer levels, and thrombocyte levels), which currently comprise the mainstay scoring system for diagnosing DIC, including for sepsis-induced coagulopathy [23]. The included albumin level data were also those assessed before DIC diagnosis, ranging from a point in time before sepsis diagnosis up to the point of sepsis diagnosis.

Coagulative imbalance may also have developed as a result of liver disease alone, i.e., before patients became septic. This is also a limitation of the study; this limitation can be addressed via a prospective study that examines serial liver function (AST, ALT, albumin, and globulin) and hemostatic parameters (D-dimer, prothrombin time, and thrombocyte level) before the patient is diagnosed with sepsis, at the time of sepsis, and at the time of DIC diagnosis. Based on our study results, we are only able to provide a general description regarding the temporal relationship of DIC development in septic patients with liver disease according to the level of thrombocytes at time of sepsis diagnosis (Table 5). In 4 out of 11 patients with liver disease and sepsis, the development of DIC was already certain after the diagnosis of sepsis, considering the normal thrombocyte level at the time of sepsis diagnosis. However, 7 of 11 patients arrived at the ER with clinical signs of both sepsis and DIC; thus, the hemostatic examinations were conducted at the same time as sepsis diagnosis, and their temporal relationship could not be specified. Therefore, on the basis of the results and limitations of this study, we recommend clinicians to carefully monitor septic patients that have low albumin, considering their possible higher risk of developing DIC.

We also found that respiratory infection predisposes septic patients to DIC. The respiratory system is one of the most common infection sites associated with sepsis and mortality due to sepsis. A study by Chou et al. (2020) found the lower respiratory tract is one of the three sites most commonly infected in adult septic patients in the US (36.6% out of 7,860,687 patients) [24]. In Indonesia, where this study was conducted, the lower respiratory tract is also the most prevalent site of infection in septic patients according to the national reimbursement plan [25]. This is in accordance with our study, where we found that in the majority of the patients (73.8%), respiratory infection was the cause of sepsis considering infections in other organ systems. The respiratory infections encountered in this study were community-acquired pneumonia, hospital-acquired pneumonia, and tuberculosis. Pathophysiologically, pneumonia is associated with activation of the clotting system and inhibition of anticoagulant factors, which are observed in both septic and non-septic patients [26]. In a study by Vail et al., an increase was also observed in protein C and markers of thrombosis (D-dimer, TAT, and F1.2), fibrinolysis (plasminogen and PAI-1), and inflammation (IL-6 and TNFa) in patients with severe sepsis as a result of community-acquired pneumonia [27].

The relationship between time before administering of antibiotics and DIC development in septic patients has not previously been extensively studied. Only one study by Kelm et al. (2013), which assessed the time of antibiotic administration in septic shock patients, revealed a statistically significant association with DIC development (OR 1.07, 95% CI 1.03–1.12, *p* < 0.001) [28]. The current surviving sepsis guidelines, from 2021, recommend that empiric antibiotics be given within the first 1 h in septic patients to reduce sepsis-associated mortality [29]. Although a direct causational explanation for the association between early antibiotic administration and reduced DIC development is lacking, early use of antibiotics may aid in reducing the number of pathogens or reduce proliferation of pathogens, thereby reducing the induction of systemic coagulation associated with systemic inflammatory and immune responses [28].

Variables found to be significant in the bivariate analysis, despite their lack of significance according to the multivariate analysis, should also be considered as potential risk factors due to their clinical and pathophysiological correlation. These factors were age ≥60 years, hematological cancer, and metabolic disease, with hematological cancer showing the highest OR of 3.286.

In their study, Kelm et al. (2013) found that DIC tends to occur in younger septic patients, although the association between the two was not statistically significant (OR 0.98; 95% CI 0.96–1; *p* = 0.19) [28]. We also did not find any association between age less than 60 years and DIC development in septic patients in our results. However, pathophysiologically, the correlation between age and DIC in septic patients may be explained through the concept of immunothrombosis, where a greater adaptive immune response, innate immune response, and inflammatory response in sepsis lead to a greater likelihood of the immune system inducing coagulation [30,31]. Activation of coagulation is mainly due to the activation of tissue factors through the pathway of pathogen-associated molecular patterns, damage-associated molecular patterns, neutrophil extracellular net, and expression of procoagulant factors on the damaged endothelium [30,31]. Meanwhile, in geriatric patients, it was found that there was a decrease in the response of the adaptive immune system and the innate immune system in terms of antigen production and expression in immune cells, which may reduce the risk of DIC [32,33].

The association between metabolic disease and DIC in sepsis cases may be pathophysiologically explained. Generally, diabetic patients are in a prothrombotic and procoagulant state with increased activation of thrombocytes, increased activation of procoagulant factors (fibrinogen, tissue factor, factor VII, and PAI-1), and resistance of plasminogen to lysis through oxidation and glycosylation of fibrinogen and plasminogen [34,35]. The proinflammatory state of septic patients with diabetes may also contribute the risk of thrombosis through immune activation [34,35]. These patients exhibit increased vascular inflammatory responses through free-radical production and a reduction in antioxidants as well as activation of NF-κB signaling pathways through fatty acid binding with Toll-like receptors [34,35]. Chemotactic and phagocytic attributes of neutrophils toward platelet-activating factors are also altered in these patients [34,35].

The relationship between hematological cancer and the incidence of DIC in sepsis can also be explained through the prohemostatic condition caused by cancer cells [36,37]. The general relationship between hematological cancer and DIC was described in a study conducted by Levi et al. (2019), which found that DIC occurs in 15–20% of hematological cancers, especially in patients with acute promyelocytic leukemia (AML M3) [23]. Tumor cells can express procoagulant molecules on their surface, including tissue factor, a major inducer of coagulation in infectious conditions, through binding to clotting factor VIIa, factor IX, and factor X [36,37]. In addition, cancer cells can also induce the action of plasminogen activator [36,37]. However, differentiating hematological cancer as a cause of DIC before patients develop sepsis or as a predisposing factor to developing DIC in septic patients remains a challenge in clinical practice, with no current study specifically addressing this matter.

Due to the design of this study, it was not possible to accurately differentiate DIC caused by cancer from DIC in septic patients predisposed by hematological cancer. This would require study with a prospective cohort design and assessing hematological malignancy in two distinct groups of non-septic patients and septic patients with hematological malignancy. Furthermore, complete hemostatic parameters to assess DIC (D-dimer, fibrinogen, thrombocyte, and prothrombin time) before a sepsis diagnosis and at the time of DIC diagnosis are recommended toward more effectively assessing the relationship between hematological cancer and DIC.

This retrospective study could only describe hematological cancer as a predisposing factor for DIC in sepsis through hematological parameters assessed at the time of sepsis diagnosis and at the time of DIC diagnosis, as complete hemostatic parameters were not determined serially due to clinical importance and patient consideration of costs. There were ten septic patients with hematological cancer who met the overt DIC criteria based on ISTH (Supplementary Table S2). Out of 10 septic patients with hematological cancer who developed DIC, 8 had low levels of thrombocyte (below 100,000/µL) and 2 had normal thrombocytes at the time of sepsis diagnosis. The patients with normal initial thrombocyte levels and 5 out of 8 patients with low initial thrombocyte levels subsequently experienced decreases in thrombocyte levels when they developed DIC, while 2 patients experienced increases in thrombocyte levels. The one remaining patient with low initial thrombocyte levels was diagnosed with DIC at the same time as sepsis, as hemostatic parameters were conducted at the time of sepsis diagnosis due to clinical signs of DIC upon arrival. Hemostatic parameters in this patient were not serially evaluated because DIC was already diagnosed, and the patient died 5 days after arrival.

Through the case-by-case description mentioned above, an assumption that hematological cancer predisposes to DIC development and worsening hematological status in septic patients could be made, considering that the majority of patients assessed (7 out of 10) experienced decreases in thrombocyte levels after sepsis, which led to DIC development.

According to the type of hematological cancer in septic patients who developed DIC, there were 3 patients with acute myelogenous leukemia, 2 patients with chronic myelogenous leukemia, 2 patients with non-Hodgkin’s lymphoma, and 2 patients with Hodgkin’s lymphoma (Supplementary Table S2). DIC in hematological cancer could develop spontaneously at the time of diagnosis, on initiation of chemotherapy [36]. Levi reported that DIC developed in 95% of AML patients at the time of diagnosis [36]. Meanwhile, a study by Chi et al. assessing DIC incidence in 236 NHL patients found that only 11.2% of patients developed DIC [38]. The results of these previous studies showed varying DIC incidence according to hematological cancer type and, therefore, must be explored further. However, up until now, there has been no study assessing the development of DIC caused by hematological cancer excluding septic patients; therefore, an accurate depiction of DIC incidence, whether caused by hematological cancer itself or sepsis, is still lacking.

## 8. Study Limitation

A limitation of this retrospective study is that it gathered only limited examination data required to fully assess SOFA score, as full examinations are not routinely conducted in daily practice, especially in a non-ICU setting. Therefore, this study used qSOFA, a simplification of SOFA score, which performs sufficiently well compared with SOFA and LODS in a non-ICU setting [18]. Since this study was a retrospective cohort study of sepsis assessed in a non-ICU setting (emergency room or ward) with limited resources, the qSOFA score was deemed sufficient and simpler for this assessment, although there is still the possibility that non-septic patients were included in the study.

Prospective studies are needed to further analyze the factors associated with DIC, especially in hematological cancer and liver disease patients. Due to its retrospective design, this study was limited regarding the ability to control serial hematological and hemostatic examinations at certain times of diagnoses; therefore, we were unable to assess the temporal aspect of DIC development. For example, in patients with hematological cancer, poor hematologic parameters such as low thrombocyte levels may be caused by the development of hematological cancer but could masquerade as DIC. Thus, not all hematological alterations may have been due to DIC.

This study sets a foundation and future directions for analyzing factors associated with DIC development in sepsis, and we recommend further prospective research on these variables. We recommend conducting a prospective cohort study evaluating complete hematologic and hemostatic parameters to assess DIC before the diagnosis of sepsis, at the time of sepsis or DIC diagnosis, and after the diagnosis of DIC.

## 9. Conclusions

Low albumin levels, respiratory infection, and antibiotic administration >1 h are associated with DIC development in septic patients. Further studies, especially multicenter studies, are needed to confirm the findings of this study.

## Figures and Tables

**Table 1:** Descriptive analysis of variables. SD, standard deviation. AST, aspartate transaminase. ALT, alanine transaminase. eGFR, estimated glomerular filtration rate. PT, prothrombin time. PTT, partial thromboplastin time.

Variables	Developed Disseminated Intravascular Coagulation (*n*: 50)	Did Not Develop Disseminated Intravascular Coagulation (*n*: 198)
Mortality, *n* (%)	38 (76%)	133 (67.2%)
Age, years, mean ± SD	49.76 ± 13.97	55.61 ± 15.20
Male, *n* (%)	25 (50%)	102 (51.5%)
Hemoglobin, g/dL, mean ± SD	8.99 ± 2.68	9.83 ± 2.57
Anemia, *n* (%)	45 (90%)	166 (83.8%)
Leukocyte, /mm^3^ median (min–max)	16,870 (10.3–163,340)	14,995 (6.6–98,860)
Leukocytosis, *n* (%)	39 (78%)	154 (77.8%)
Neutrophil/lymphocyte ratio, median (min–max)	10.49 (0.06–91.7)	13.39 (0.03–107.6)
High neutrophil lymphocyte ratio, *n* (%)	26 (52%)	131 (66.2%)
Thrombocyte, /mm^3^ median (min–max)	75,500 (2000–625,000)	246,000 (439–855,000)
AST, U/L, median (min–max)	45.5 (6–6859)	38 (6–2143)
ALT, U/L, median (min–max)	31 (6–1632)	23 (6–390)
Albumin, g/dL, median (min–max)	2.42 (1.29–4)	2.55 (1.46–4.67)
Albumin ≤2.5 g/dL, *n* (%)	30 (60%)	85 (42.9%)
Ureum, mg/dL, median (min–max)	69.15 (18–298.2)	69.35 (7.2–251)
Creatinine, mg/dL, median (min–max)	1.25 (0.3–11.2)	1.5 (0.1–15)
eGFR, mL/min/1.73 m^2^ median (min–max)	55.05 (4–170)	48.6 (3–261)
eGFR <60 mL/min/1.73 m^2^, *n* (%)	26 (52%)	113 (57.1%)
PT, seconds, median (min–max)	14.85 (10.1–44.2)	12.1 (9–120)
PTT, seconds, median (min–max)	49.7 (29.3–180)	41.6 (19.3–190)
D-dimer, ng/mL, median (min–max)	8535 (400–52,250)	3254 (100–352,000)
Fibrinogen, mg/dL, median (min–max)	231.6 (38–758)	442 (38–1044)
Pulmonary infection, *n* (%)	41 (82%)	142 (71.7%)
Solid cancer, *n* (%)	19 (38%)	65 (32.8%)
Hematological cancer, *n* (%)	10 (20%)	14 (7.1%)
Metabolic disease, *n* (%)	11 (22%)	79 (39.9%)
Autoimmune disease, *n* (%)	2 (4%)	13 (6.6%)
Liver disease, *n* (%)	11 (22%)	37 (18.7%)
Heart failure, *n* (%)	3 (6%)	21 (10.6%)
Chronic kidney disease, *n* (%)	26 (52%)	113 (57.1%)
Received hemodialysis, *n* (%)	15 (30%)	56 (28.3%)
Received antiplatelet, *n* (%)	2 (4%)	10 (5.1%)
Positive Blood culture, *n* (%)	11 (22%)	44 (22.2%)
Received antibiotic ≥ 1 h, *n* (%)	39 (78%)	124 (62.6%)

**Table 2:** Site of infection (*n* = 248).

Site of Infection	*n* (%)
Pulmonary system	183 (73.8%)
Gastrointestinal system	20 (8.1%)
Hepatobiliary system	10 (4%)
Nervous system	2 (0.8%)
Reproductive system	1 (0.4%)
Kidney and urinary system	11 (4.4%)
Soft tissue	21 (8.5%)

**Table 3:** Hematological cancer type (*n* = 24). AML, acute myeloid leukemia; ALL, acute lymphoblastic leukemia; CML, chronic myeloid leukemia; CLL, chronic lymphocytic leukemia.

Hematological Cancer Type	*n* (%)
AML	5 (20.8%)
ALL	1 (4.2%)
CML	2 (8.3%)
CLL	0 (0%)
Myeloproliferative neoplasm	1 (4.2%)
Hodgkin’s lymphoma	2 (8.3%)
Non-Hodgkin’s lymphoma	10 (417%)
Multiple myeloma	3 (12.5%)

**Table 4:** Bivariate analysis of the association between variables and DIC development in septic patients.

Variables	Odds Ratio	95% Confidence Interval	*p*-Value
Male	0.941	0.506–1.750	0.848
Female	Reference		
Age ≥60 years	0.467	0.234–0.933	0.031
Age <60 years	Reference		
Respiratory infection	1.797	0.819–3.939	0.144
No respiratory infection	Reference		
Solid cancer	1.254	0.659–2.387	0.490
No solid cancer	Reference		
Hematological cancer	3.286	1.362–7.926	0.008
No hematological cancer	Reference		
Metabolic disease	0.425	0.205–0.879	0.021
No metabolic disease	Reference		
Albumin ≤2.5 g/dL	1.994	1.060–3.751	0.032
Albumin >2.5 g/dL	Reference		
Liver disease	1.227	0.575–2.621	0.597
No liver disease	Reference		
Autoimmune disorder	0.593	0.129–2.717	0.501
No autoimmune disease	Reference		
Heart failure	0.538	0.154–1.881	0.332
No heart failure	Reference		
eGFR <60 mL/min/1.73 m^2^	0.815	0.437–1.518	0.519
eGFR ≥60 mL/min/1.73 m^2^	Reference		
Chronic kidney disease	0.815	0.437–1.518	0.519
No chronic kidney disease	Reference		
Received hemodialysis	1.087	0.551–2.144	0.810
Did not receive hemodialysis	Reference		
Received antiplatelet	0.783	0.166–3.694	0.758
Did not receive antiplatelet	Reference		
Anemia	1.735	0.639–4.709	0.279
No anemia	Reference		
Leukocytosis	1.013	0.479–2.141	0.973
No leukocytosis	Reference		
High neutrophil lymphocyte ratio	0.554	0.296–1.038	0.065
Low neutrophil lymphocyte ratio	Reference		
Positive blood culture	0.987	0.467–2.086	0.973
Negative blood culture	Reference		
Received antibiotic ≥1 h	2.116	1.021–4.384	0.044
Received antibiotic <1 h	Reference		

**Table 5:** Multivariate analysis of association between variables and DIC development in septic patients *.

Variables	Odds Ratio	95% Confidence Interval	*p*-Value
Age ≥60 years	0.523	0.247–1.107	0.090
Age <60 years	Reference		
Albumin ≤2.5 g/dL	2.363	1.201–4.649	0.013
Albumin >2.5 g/dL	Reference		
Respiratory infection	2.414	1.046–5.571	0.039
No respiratory infection	Reference		
Metabolic disease	0.485	0.224–1.051	0.067
No metabolic disease	Reference		
Received antibiotic ≥1 h	2.181	1.014–4.689	0.046
Received antibiotic <1 h	Reference		

* Adjusted for gender, solid cancer, hematological cancer, heart failure, GFR value, CKD status, hemodialysis status, antiplatelet therapy, anemia, leukocytosis, NLR, and blood culture.

## Data Availability

The data presented in this study are available on request from the corresponding author. The data are not publicly available due to privacy.

## Supplementary Materials

The following supporting information can be downloaded at https://www.mdpi.com/article/10.3390/jcm11216480/s1. Table S1. Characteristics of patients with sepsis, DIC, and liver disease; Table S2. Characteristics of patients with sepsis, DIC, and hematological cancer.
