# Supplementary material for: Disseminated Intravascular Coagulation in Sepsis and Associated Factors
Source: J Clin Med. 2022 Oct 31;11(21):6480. doi: 10.3390/jcm11216480 (PMC9658286; doi:10.3390/jcm11216480)
Supplement: Supplementary file 1 [file jcm-11-06480-s001.zip › jcm-1972620-supplementary.pdf]

Supplementary Materials

Table S1. Characteristics of patients with sepsis, DIC, and liver disease.

Patient	Thrombocyte Level on Sepsis Diagnosis	Thrombocyte Level on DIC Diagnosis	Diagnosis of Liver Disease	Temporal Relation of DIC to SEPSIS
A	158,000	36,000	Unspecified	DIC developed after sepsis
B	112,000	223,000	Congestive liver disease	DIC developed after sepsis
C	625,000	79,000	Liver cirrhosis	DIC developed after sepsis
D	129,000	30,000	Cholangitis	DIC developed after sepsis
E	428,000	428,000	Liver cirrhosis	DIC and sepsis diagnosed at the same time
F	144,000	144,000	Liver cirrhosis	DIC and sepsis diagnosed at the same time
G	227,000	227,000	Hepatoma	DIC and sepsis diagnosed at the same time
H	48,000	48,000	Hepatitis C	DIC and sepsis diagnosed at the same time
I	37,000	37,000	Hepatocellular carcinoma	DIC and sepsis diagnosed at the same time
J	51,000	51,000	Liver failure	DIC and sepsis diagnosed at the same time
K	67,000	65,000	Hepatitis C, liver cirrhosis	DIC developed after sepsis or pre-existed before sepsis

Table S2. Characteristics of patients with sepsis, DIC, and hematological cancer.

Patient	Thrombocyte Level on Sepsis Diagnosis	Thrombocyte level on DIC Diagnosis	Haematological Cancer Type based on Medical Record
A	13,000	6000	Acute Myelogenous leukemia (M3)
B	33,000	15,000	Acute Myelogenous leukemia unspecified
C	9000	36,000	Chronic Myelogenous Leukemia
D	14,000	14,000	Non-Hodgkin's Lymphoma
E	13,000	22,000	Chronic Myelogenous Leukemia
F	62,000	5000	Acute myelogenous leukemia (M2)
G	224,000	119,000	Hodgkin's lymphoma
H	33,000	29,000	Hodgkin's Lymphoma
I	88,000	20,000	Non-Hodgkin's Lymphoma B Cell CD20 minimal IIIE with pancreatic involvement
J	310,000	23,000	Acute Lymphocytic leukemia
